# The Natural History of Alcoholism

**Published:** 1996

**Authors:** George E. Vaillant, Susanne Hiller-Sturmhöfel

**Affiliations:** George E. Vaillant, M.D., is a professor of psychiatry at Harvard Medical School, director of the Study of Adult Development at the Harvard University Health Services, and director of research in the Division of Psychiatry at Brigham and Women’s Hospital, Boston, Massachusetts. Susanne Hiller-Sturmhöfel, Ph.D., is a science editor for Alcohol Health & Research World

**Keywords:** AOD dependence, disease course, longitudinal study, prospective study, risk factors, etiology, familial alcoholism, sociocultural AODC (causes of AOD use, abuse, and dependence), family environment, antisocial behavior, emotional and psychiatric depression, college student, urban area, heavy AOD use, AOD abstinence, Alcoholics Anonymous, treatment goals

## Abstract

Over the past 55 years, two longitudinal studies have been monitoring the drinking behaviors and their consequences of several hundred men from adolescence and early adulthood to old age. The studies identified co-occurring sociopathy, cultural factors (e.g., ethnicity), and genetic factors (i.e., a family history of alcoholism) as risk factors for alcoholism. n most alcoholics, the disease had a progressive course, resulting in increasing alcohol abuse or stable abstinence. However, some alcoholics exhibited a nonprogressive disease course and either maintained a stable level of alcohol abuse or returned to asymptomatic drinking. Long-term return to controlled drinking, however, was a rare and unstable outcome. Formal treatment, with the exception of attending Alcoholics Anonymous, did not appear to affect the men’s long-term outcomes, whereas several non-treatment-related factors were important for achieving stable recovery.

In many ways, alcoholism differs from most other diseases. First, it generally develops slowly over a person’s life and can occur in people of all ages. Second, it has no single known cause: Heredity, culture, economics, and the environment all contribute to its development, and each alcoholic has his or her own personal drinking history. Third, both alcoholics and their alcohol-related disabilities can change over time. For example, alcohol can have long-term effects on the central nervous system that may alter an alcoholic’s personality and perception of the past. Finally, no known cure exists; although some patients recover either “spontaneously” or after treatment, many patients never recover. In an effort to increase our understanding of alcoholism and develop more effective prevention and treatment programs, alcohol researchers are studying the development and course of the disease in people of various ages.

Several different methodological approaches can be used for investigating alcoholism and its characteristics, including cross-sectional and longitudinal studies. Cross-sectional studies examine large numbers of subjects of various ages and social backgrounds representative of the general population. Longitudinal studies, in contrast, usually include smaller and less representative samples, but the subjects are followed over longer periods (e.g., up to 50 years) and reexamined repeatedly. Thus, although the overall sample may be biased, the disease progress in each person can be documented in more detail.

Alcohol studies also vary by whether the subjects are evaluated retrospectively or prospectively. In retrospective studies, researchers select subjects with a specific disorder (e.g., alcoholism) and, using interviews, medical records, and other sources of information, try to determine the factors that contributed to the disease’s development. Conversely, the subjects of prospective studies are disease free at the study’s outset; accordingly, some subjects will develop the disorder under investigation, whereas others will not. This approach allows researchers to analyze the premorbid characteristics of both groups of subjects.

Most analyses of the development and course of alcoholism have used a cross-sectional, retrospective design, with researchers recruiting alcoholics (e.g., from treatment facilities) and establishing their drinking histories. This approach may not always produce reliable results, however, because alcoholism is a chronic disease that changes in its severity and manifestations over time. Consequently, chronic alcohol consumption may gradually alter an alcoholic’s personality. Furthermore, guilt, misattribution, and the passage of time can cause unwitting misrepresentation of an alcoholic’s characteristics before disease onset. Longitudinal, prospective studies therefore are better suited for analyzing alcoholism’s development, determining the subjects’ premorbid states, and monitoring alcohol-induced changes.

This article summarizes findings from two longitudinal, prospective studies (now coordinated at Harvard Medical School) that have been following two groups of men from about 1940 to the present (Vaillant 1995). By integrating these data with other research findings obtained over the years, this article attempts to provide some answers to the following questions:

- Is alcoholism an independent disease or the symptom of an underlying disorder?
- Do certain characteristics distinguish people who eventually become alcoholics from those who do not?
- Is alcoholism always a progressive disorder?
- How does alcoholism treatment or participation in self-help groups, such as Alcoholics Anonymous (AA), influence the disease process?
- Is abstinence the only reasonable treatment goal, or can alcoholics safely return to social drinking?

## The Study Samples

Most findings discussed in this article were derived from two groups of men—referred to as the “College” sample and the “Core City” sample—who have been studied for more than 50 years by researchers at the Harvard University Health Services. (Unless otherwise noted, these findings are summarized in Vaillant 1995.) Neither one of the two ongoing studies has focused solely on alcohol and other drug abuse. The initial goal of the College study was to investigate the development of “healthy” college students, whereas the Core City sample originally served as a control group in a study of juvenile delinquents. Because both studies have used a multidisciplinary and comprehensive approach, however, they also provide information about the development of alcoholism and related disorders. Moreover, although neither sample is representative of the general population (i.e., they include only Caucasian men and primarily represent two extremes on the socioeconomic scale), the combined data provide some insight into various factors contributing to the natural history of alcoholism.

### The College Sample

The 268 participants in the College sample were recruited from the sophomore classes at Harvard University between 1939 and 1944 and represented about 7 percent of each class. Whereas 10 percent of the sample were randomly selected, the other 90 percent fulfilled the selection criterion of being “sound” students (i.e., academically successful and experiencing no medical or psychological problems). Because of this selection criterion, the College sample generally comprised socioeconomically privileged men who were likely to lead successful, “satisfactory” lives. They attained an average of 18 years of education.

At the beginning of the study, a psychiatrist extensively interviewed each participant to determine his family background, career plan, and values. A social worker then interviewed each subject to establish his social history. By visiting each subject’s parents, the social worker also obtained information about the subject’s childhood development and compiled the family’s history (comprising information on the grandparents, aunts, uncles, and first cousins) of mental and physical illnesses, including alcoholism. Finally, each study participant underwent a thorough physical examination, several physiological tests, and several psychological tests measuring intelligence.

After graduating from college, the men received extensive questionnaires annually until 1955 and every 2 years since then. These questionnaires addressed health, family situation, employment, habits (e.g., alcohol consumption, smoking, and sports activities), and political views. Each subject’s health also was assessed by complete physical examinations every 5 years from age 45 to age 70. All men were reinterviewed in 1951. In addition, 50 percent were reinterviewed at about age 47 and the other 50 percent at about age 55. Furthermore, all men with suspected alcohol problems were reinterviewed between 1971 and 1976.

### The Core City Sample

The 456 men of the Core City sample were selected between 1940 and 1944, at ages 11 to 16, from Boston inner-city schools as a control group for a sample of juvenile delinquents (i.e., they were matched to the delinquents by age, intelligence, neighborhood crime rate, and ethnicity). The participants predominantly came from lower social classes and attained an average of only 11 years of education. In addition, the Core City subjects had a variety of ethnic backgrounds, and 61 percent of their parents were immigrants.

Similar to the College sample, the Core City subjects were studied by physicians, psychologists, psychiatrists, and social workers. The initial assessment included interviews with the boys, their families, and their schools as well as a review of information from public records (e.g., through the criminal justice system, social services, and mental health services). Based on this information, the investigators documented the subjects’ family histories of criminality, mental health, and alcoholism for three generations. The Core City participants were subsequently reinterviewed at ages 25, 31, and 47; again, concurrent searches were conducted of public records and of data from mental health agencies, hospitals, and law enforcement agencies. Beginning in 1974, the men were followed with questionnaires every 2 years and physical examinations every 5 years.

## The Development of Alcoholism

Before examining how alcoholism develops, one must first define what alcoholism is. For the purpose of this article, the term “alcoholism” encompasses two diagnoses: alcohol abuse and alcohol dependence. These diagnoses were determined according to the criteria of the *Diagnostic and Statistical Manual of Mental Disorders, Third Edition* (DSM–III) (American Psychiatric Association 1980). Both definitions imply that alcoholism is not characterized by one specific feature but requires the clustering of several alcohol-related symptoms. For example, study participants who were diagnosed as alcohol dependent according to the DSM–III criteria generally also experienced at least 8 of the 16 alcohol-related problems listed in the Problem Drinking Scale (PDS),^1^ another tool used to define alcoholism. Thus, alcoholism can be viewed as one end of a continuum of drinking behaviors ranging from social drinking to severe dependence.

### The Etiology of and Risk Factors for Alcoholism

One important question addressed by alcohol researchers focuses on the mechanisms underlying alcoholism’s development (i.e., the etiology): Is alcoholism an independent disorder that can lead to psychiatric symptoms, or is it a symptom or consequence of an underlying psychiatric disorder (e.g., depression, sociopathy, or neurosis)? Prospective longitudinal studies are particularly well suited to address this question, because they begin following subjects before the onset of a disorder. Prospective studies thus enable researchers to establish the temporal order in which two diseases (e.g., alcoholism and depression) occur. Other lines of research examine risk factors for alcoholism that exist early in life and that might help identify people who are likely to become alcoholic. The Harvard University Health Services studies focused on four such potential influences: sociopathy (i.e., antisocial personality disorder), cultural factors (e.g., the subject’s ethnic background), genetic factors, and childhood environment.

### The Role of Psychiatric Disorders

Most studies investigating associations of alcoholism with psychiatric disorders have focused on depression, because both alcoholism and depression tend to run in families and frequently occur together in the same person (Merikangas and Gelernter 1990). For example, in the College sample, severe depression was five times more common among alcoholics than among nonalcoholics. The common comorbidity of alcoholism and depression has led to the hypothesis that people begin to drink to self-medicate, or alleviate, their depression (i.e., alcoholism is secondary to depression). Several lines of evidence, including the following, indicate, however, that in most cases depression is a consequence of alcoholism:

- The rate of alcoholism among manic-depressive patients, who according to the self-medication hypothesis should use alcohol frequently, is not higher than the rate among other psychiatric patients (Woodruff et al. 1973; Morrison 1974).
- Biochemical tests that can distinguish between genetically determined (i.e., primary) and environmentally determined (i.e., secondary) depression found that depressed alcoholics more closely resembled patients with evidence of environmentally induced depression than did patients with genetically determined depression (Schlesser et al. 1980).
- Data from the College sample indicated that subjects who later became alcoholic did not manifest, either as children or as adolescents, the personality or childhood characteristics that would have predisposed them to depression (Vaillant 1980). Moreover, of the 14 College men with both alcoholism and depression, only 4 experienced depression before they became alcoholic, a proportion that can be attributed to chance alone (Vaillant 1995).
- Although depression and alcoholism may run in the same families, multigenerational studies have documented that the predispositions for both disorders are genetically separate (Weissman et al. 1984; Merikangas and Gelernter 1990).
- In clinical studies, the use of antidepressants to treat patients with both alcoholism and depression did not alter the course of alcoholism; however, abstinence from alcohol in such patients alleviated the depression (Brown et al. 1988; Dorus et al. 1989; Mason et al. 1996).

Researchers reached similar conclusions when investigating the associations of alcoholism with other disorders (e.g., generalized anxiety disorder). Thus, in most patients, alcoholism appears to be an independent disorder that does not develop secondary to other psychiatric disorders, such as depression. The one psychiatric disorder that clearly contributes to the risk for alcoholism is sociopathy.

#### Sociopathy

Several studies have indicated that sociopathy^2^ is a predisposing factor for alcoholism (e.g., Cloninger et al. 1988). The Core City study’s prospective design allowed researchers to determine whether sociopathy could lead to alcoholism (i.e., whether alcoholism was a symptom of the underlying behavioral disorder) or whether sociopathic symptoms were a consequence of alcoholism. Although the Core City subjects were originally selected because they did not exhibit antisocial behavior, 25 subjects eventually met the minimal criteria for sociopathy. These subjects were four times more likely to become alcohol dependent than the subjects who did not develop antisocial behavior; the rates of alcohol abuse, however, did not differ between the two groups. Moreover, the age at onset of alcoholism was significantly lower for the sociopathic subjects than for those who were nonsociopathic. Because most sociopathic alcoholic subjects developed sociopathic symptoms before they abused alcohol, alcoholism can be considered a symptom of underlying sociopathy. The overall proportion of sociopathic subjects among the alcoholics, however, was small. Thus, although many sociopaths abuse alcohol as part of their antisocial behavior, most alcoholics are not sociopathic except as a result of their addiction.

#### Cultural Factors

The Core City sample was particularly useful for studying the influences of ethnic background on drinking behavior, because the parents of more than 60 percent of the subjects were born outside the United States. Thus, the Core City sample represented a variety of ethnic backgrounds, including Irish, Polish, Russian, English, Northern European, Italian and other Southern European, Anglo Canadian, and French Canadian.

When comparing the lifetime rates of abstinence, social (i.e., asymptomatic) drinking, alcohol abuse, and alcohol dependence among men with different ethnic backgrounds, the researchers found that the alcoholism rates varied among the ethnic groups, although a similar proportion of men in each group (about 20 percent) were abstinent. Most strikingly, alcohol abuse and dependence were five times less common in men of Italian and other Southern European descent compared with other ethnic groups (e.g., Irish).

These differences may be attributed, at least in part, to variations in the cultural attitudes toward alcohol consumption (e.g., Heath 1975; Greely and McReady 1980). For example, in the Italian culture, wine is consumed regularly with meals, and children usually are introduced to alcohol by their parents. Although alcohol consumption is sanctioned even among children, moderation is encouraged and intoxication is proscribed. Conversely, alcohol consumption in Ireland is illegal before age 21, and drinking mainly occurs in bars and not within the family or at meals. Moreover, in Ireland, drunkenness in men is socially sanctioned—“a good man’s failing.” Although these examples oversimplify the many factors determining ethnic variance in alcohol consumption patterns, they suggest that cultural practices contribute to the risk for alcoholism.

#### Genetic Factors

A genetic predisposition (i.e., a family history) to alcoholism is a well-known risk factor, and many prospective and retrospective studies have demonstrated that the children of alcoholic parents—particularly sons of alcoholic fathers—are at increased risk of becoming alcoholic compared with children whose parents are not alcoholic (e.g., Schuckit 1995). Both the College and Core City samples confirmed these observations. In the College sample, 26 percent of the men with alcoholic relatives, but only 9 percent of the men without alcoholic relatives, became alcoholics themselves. In the Core City sample, the corresponding numbers were 34 percent and 10 percent, respectively.

It is difficult, however, to separate genetic and environmental effects of alcoholic family members on the development of alcoholism in a study subject. Frances and colleagues (1980) observed that alcoholics with family histories of alcoholism were more likely than alcoholics without such family histories to come from severely disrupted families, to exhibit antisocial behavior, and to have performed less well in school. Thus, living in a household with an alcoholic family member can potentially cause an environmental disruption that may increase the risk for alcoholism. These environmental effects, however, do not account for the entire risk associated with a family history of alcoholism. For example, 29 percent of the Core City men with alcoholic ancestors (i.e., family members who were not part of the subject’s environment) became alcoholic, whereas only 14 percent of the men without alcoholic ancestors developed alcoholism. (For an extensive review of the genetics of alcoholism, see *Alcohol Health & Research World* vol. 19, no. 3.)

#### Childhood Environment

When examining factors that might predispose a person to alcoholism, researchers also have investigated subjects’ childhood environments. This approach includes evaluating both environmental strengths and weaknesses, such as family cohesiveness, degree of parental supervision, relationship of the child with the parents, and home atmosphere. Early studies found that unstable childhoods with broken homes and inconsistent upbringing seemed to predict future alcoholism (McCord and McCord 1960; Robins 1966). Superficial analysis of the Core City sample appeared to confirm these findings. “Warm” and cohesive environments and close relationships were most characteristic of the men who did not become alcoholics. Further analysis showed, however, that these differences generally could be accounted for by the presence or absence of an alcoholic parent in the subject’s family. Men with few childhood environmental weaknesses but an alcoholic parent (who, in fact, they might not live with) were four times more likely to become alcoholic themselves than men with many childhood environmental weaknesses—and perhaps an alcoholic stepparent—but no alcoholic parent. Accordingly, if alcoholism in biological parents is controlled for, a troubled childhood environment per se does not appear to affect a person’s risk for alcoholism, a finding that was confirmed in the College sample.

In summary, the analyses of the College and Core City samples found that both cultural and genetic factors can predispose a person to alcoholism, whereas childhood environment per se plays a much less significant role. Furthermore, although alcoholism generally is not the consequence or symptom of an underlying psychiatric disorder, an antisocial personality may lead to alcoholism. Some of these findings contrast with previous retrospective studies that found associations between psychiatric disorders, such as depression, and alcoholism. These discrepancies may be explained by differences in the study design. The results of retrospective studies sometimes may be misleading, because prolonged alcohol abuse can impair or distort alcoholics’ recollections of their childhood and adolescence as well as their ability to distinguish cause from effect (i.e., the temporal sequence of the development of certain symptoms).

## The Course of Alcoholism

As stated in the article’s introduction, alcoholism generally develops over long periods of time. Furthermore, although the disorder progresses continuously in some alcoholics, it remains stable or even regresses spontaneously in others. Because the disease course varies widely among individual alcoholics, longitudinal studies that repeatedly examine the same subjects are especially well suited for investigating patterns of alcohol consumption and the development of alcoholism over time.

### Patterns of Alcohol Consumption

**Figure 1:**
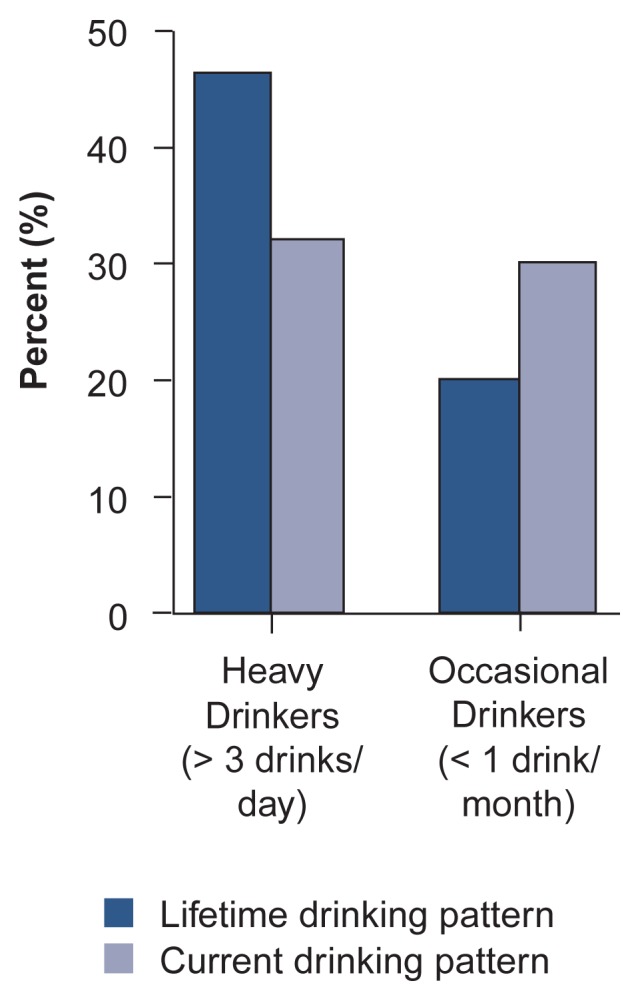
Selected current and lifetime drinking patterns of Core City subjects evaluated at age 47. The data suggest that alcohol consumption stabilizes or declines during middle age. SOURCE: Vaillant 1995.

To determine changes in the patterns of alcoholism, the Core City subjects were categorized at ages 47 and 60 according to their DSM–III diagnoses for lifetime alcohol abuse and dependence. This comparison found that only 9 of the 260 men who were nonalcoholic at age 47 had developed alcohol abuse or dependence at age 60. Moreover, none of the 59 men who were classified as alcohol abusers at age 47 had progressed to alcohol dependence at age 60. Thus, alcoholism does not appear to be an inevitably progressive disease, and alcohol consumption tends to stabilize or decline during middle and older age. A similar trend was evident when the men’s lifetime drinking patterns were compared with their current patterns at age 47 (figure 1): Although nearly one-half of the men evaluated had been heavy drinkers (i.e., consumed three or more drinks per day) at some point during their lifetime, only approximately one-third still drank that much at age 47. Conversely, more men consumed less than one drink per month at age 47 than had a lifetime pattern of this level of alcohol consumption. These observations suggest that some heavy drinkers and even some alcohol abusers will maintain their drinking patterns or reduce them with increasing age.

### The Evolution of Alcoholism Over Time

As previously indicated, alcoholism does not progress inexorably in all patients, and possible long-term outcomes of alcoholism include a return to abstinence; a return to controlled, or asymptomatic, drinking; and continued alcoholism. In part, outcome depends on the alcoholic’s personal characteristics, such as age. For example, an 8-year followup study of relatively young alcoholic prisoners (i.e., average age of 27) found that one-third of the subjects returned to asymptomatic drinking during the study (Goodwin et al. 1971). The subjects most commonly cited marriage and/or an increase in family responsibilities as reasons for their change in drinking behavior. These findings, which also were confirmed in the Core City sample, suggest that in young alcohol abusers who have not yet developed alcohol dependence, changes in their social responsibilities and peer groups often can reverse their drinking patterns. Other studies also noted that drinking behavior can change considerably between adolescence and young adulthood but tends to become more stable during middle age (Jessor 1987; Fillmore 1987).

Another common finding of longitudinal studies is that the prevalence of alcoholism declines as the subjects age. For example, an analysis of eight long-term studies demonstrated that out of 675 alcoholic subjects who were followed for an average of 15 years (until they were approximately 60 years old), only 25 percent were still alcoholic at the end of the studies (Vaillant 1995). Several factors appear to contribute to this decline. For example, several studies indicate that about 2 percent of all alcoholics return to stable abstinence each year, with or without receiving treatment. Furthermore, after age 40, roughly 2 percent of all alcoholics die each year.

#### The Core City Sample

**Figure 2:**
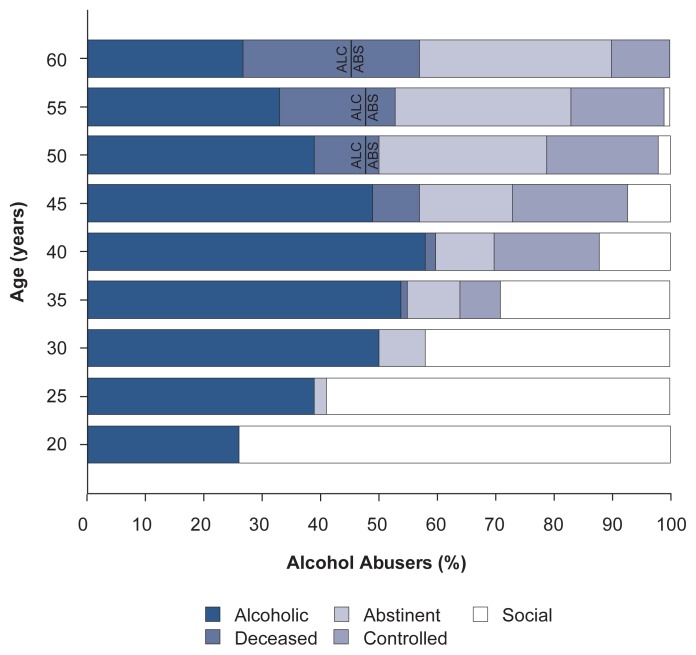
Life course of alcohol abuse of the 116 Core City men who met the DSM–III criteria for alcohol abuse at some point during the Harvard study and who remained active in the study. The proportion of men who died while active alcohol abusers is labeled “ALC”; the proportion of men who died while stably abstinent is labeled “ABS.” At age 20, nearly one-third of the men had been diagnosed as alcohol abusers; the remainder were still classified as social drinkers. By age 60, all 116 men had received their diagnoses of alcohol abuse; no social drinkers remained. Those who drank were either abusing alcohol or had returned to controlled drinking (i.e., they were able to limit their alcohol intake to the point where they no longer met the DSM-III criteria for active alcohol abuse.) SOURCE: Adapted from Vaillant 1995.

The Core City participants included 116 men who met the diagnosis for alcohol abuse and/or dependence at some points in their lives and whose life courses of alcoholism could be charted (figure 2). As previously mentioned, these men were interviewed at ages 25, 31, and 47 and received biennial questionnaires thereafter. The analyses found that the number of alcoholics increased steadily until age 40 but subsequently declined. At age 60, only 27 percent of the alcoholics were still actively abusing alcohol. Almost one-third of the alcoholic men had died before their 60th birthday, most of them as active alcoholics. Another one-third of the men were stably abstinent, and only about 10 percent had returned to asymptomatic drinking.

In most Core City subjects, the progression from social drinking to alcohol abuse and alcohol dependence occurred gradually, generally over a period of 3 to 15 years. This rate of progression can vary significantly, however, depending on the subjects studied. For example, in the College sample, progression to alcoholism often occurred even more slowly, with some subjects drinking asymptomatically for as long as 20 years before becoming alcoholic. Conversely, sociopathic alcoholics in the Core City sample exhibited a much more rapid onset of alcoholism.

When the evolution of alcoholism in individual alcoholics was studied in more detail, the subjects fell into two main categories—those with a progressive disease course and those with a nonprogressive, atypical disease course. Each category comprised two subgroups. The alcoholics with a progressive course either continued to drink, despite a worsening of their alcohol-related problems, or became stably abstinent in response to the consequences of their drinking. In contrast, atypical alcoholics either maintained a relatively stable pattern of alcohol abuse or returned to controlled drinking. When comparing the outcomes of the four groups at ages 47 and 60 (or at the time of their deaths if they had died before their 60th birthdays), the researchers obtained the following results:

- Of the 35 men with progressive alcoholism at age 47, approximately two-thirds continued to abuse alcohol at age 60. The other subjects at age 60 either became stably abstinent or returned to controlled drinking for more than 3 years.
- Of the 38 men who had been stably abstinent at age 47, the vast majority remained abstinent at age 60; only 4 men relapsed and 1 man returned to controlled drinking.
- Of the 19 men classified as atypical, nonprogressive alcohol abusers at age 47, approximately 40 percent remained alcohol abusers at age 60. Two men were abstinent at age 60, and 4 men had returned to controlled drinking.
- Of the 18 men who had returned to asymptomatic drinking at age 47, equal numbers relapsed to alcohol abuse or maintained a controlled drinking pattern at age 60. Four men became stably abstinent.

In general, the atypical alcoholics exhibited less severe alcoholism (e.g., had fewer alcohol-related problems on the PDS and were classified as alcohol abusers but not alcohol dependent by DSM–III criteria). They also had fewer risk factors for alcoholism (e.g., alcoholic relatives or behavioral problems in school) than alcoholics with a progressive disease course. Moreover, atypical alcoholics were less likely to die before their 60th birthday than progressive alcoholics. Surprisingly, among the progressive alcoholics, no significant differences either in alcoholism severity or in risk factors distinguished the men who continued to abuse alcohol and those who achieved stable abstinence.

#### The College Sample

**Figure 3:**
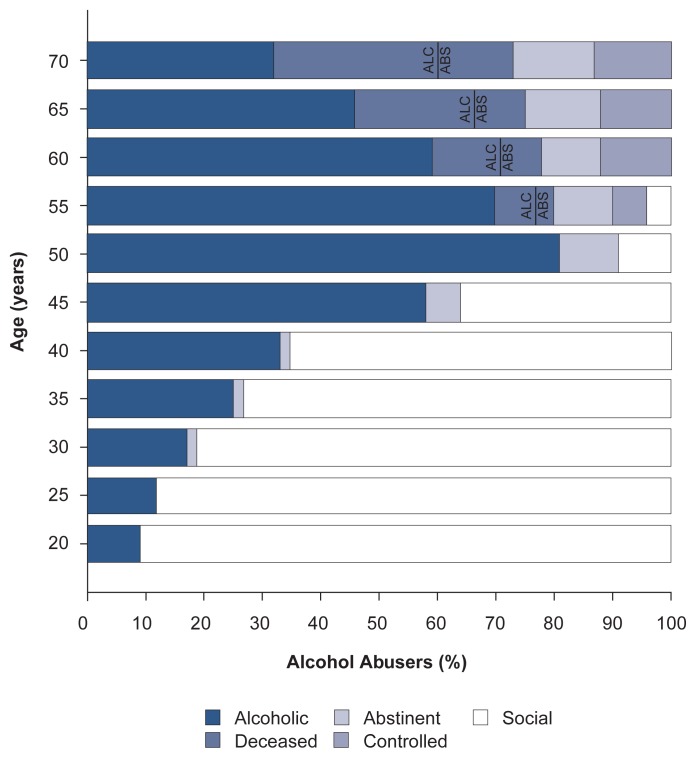
Life course of alcohol abuse of the 46 College men who met the DSM–III criteria for alcohol abuse at some point during the Harvard study and who remained active in the study. The proportion of men who died while active alcohol abusers is labeled “ALC”; the proportion of men who died while stably abstinent is labeled “ABS.” At age 20, only about 10 percent of the men had been diagnosed as alcohol abusers; the remainder were still classified as social drinkers. By age 60, all 46 men had received their diagnoses of alcohol abuse; no social drinkers remained. Those who drank were either abusing alcohol or had returned to controlled drinking (i.e., they were able to limit their alcohol intake to the point where they no longer met the DSM-III criteria for active alcohol abuse.) SOURCE: Adapted from Vaillant 1995.

In most Core City subjects, alcoholism was a progressive disease that either led to chronic, progressive alcohol abuse and dependence or required stable abstinence. In contrast, the College sample presented a very different picture of alcoholism’s evolution. As a group, the College subjects were characterized by a social environment different from that of the Core City subjects, a factor which appeared to influence their life courses of alcoholism (figure 3). For example, alcoholism developed later in life among the College subjects than among the Core City subjects: The age of onset (i.e., the age when the subjects first met the DSM–III criteria for alcohol abuse) for most of the College men was between 40 and 50 years, whereas most Core City men became alcoholic before age 30. Also, significantly fewer College alcoholics than Core City alcoholics became abstinent after age 40. Finally, alcohol abuse in most College alcoholics became neither worse nor better between the ages of 45 and 70, although many men died before their 70th birthday.

#### Clinically Treated Alcoholics

Researchers also analyzed the disease course of 100 subjects with severe alcoholism who required detoxification and therefore had entered the treatment system (Vaillant 1995). After 8 years’ followup, 34 percent of the subjects had achieved stable abstinence, 29 percent had died, and 26 percent still were abusing alcohol. Subjects who had a stable social environment (i.e., employment or a functional marriage) or who frequently went to AA meetings had the highest rates of abstinence. Thus, subjects with stable social adjustment apparently could recover on their own after receiving initial treatment, whereas subjects with social instability appeared to require frequent AA attendance to achieve abstinence. Overall, however, treatment other than AA did not significantly improve the subjects’ outcomes (i.e., the achievement of stable abstinence) compared with the untreated Core City or College subjects. In all subject groups, the rate of stable remission was between 2 and 3 percent per year.

## Recovery From Alcoholism

Recovery from alcoholism can have two possible outcomes: stable abstinence or a less stable return to controlled drinking. For both the Core City and College samples, stable abstinence was defined as the consuming of alcohol less than once per month for at least the past 3 years while living in the community (i.e., not institutionalized or imprisoned). According to these criteria, 47 of 110 Core City men (i.e., 42 percent) who had ever been classified as alcoholic and for whom sufficient data were available had achieved stable abstinence by age 60. In contrast, the number of stably abstinent alcoholics among the College men was significantly lower (10 of the 52 subjects, or 19 percent, by age 70).

To identify characteristics that could distinguish men who achieved stable abstinence from those who did not, researchers compared childhood and other risk factors, alcoholism symptoms, and other characteristics (e.g., treatment experiences) of the abstinent and nonabstinent men. These analyses found that men who did not achieve stable abstinence, compared with securely abstinent men, were more likely to have fulfilled alcohol dependence criteria and to have been binge drinkers. They were also more likely to attend AA meetings frequently. No other distinguishing characteristics existed. Moreover, with the exception of AA attendance, the subjects’ treatment experiences (e.g., treatment in a clinic, with psychotherapy, or with disulfiram) did not appear to affect long-term outcomes. These findings are consistent with other studies that found no differences in outcome whether the patients received inpatient or outpatient treatment or brief interventions (Lindström 1992; Chapman and Huygens 1988). Researchers continue to debate, however, whether AA attendance is the cause or consequence of abstinence. Finally, almost one-half of the Core City men analyzed retrospectively credited “willpower” as an integral factor in their ability to achieve abstinence. Prospective studies suggest, however, that the prospectively identified personality trait of willpower failed to predict outcome (Vaillant 1995).

### Non-Treatment-Related Factors Associated With Abstinence

To determine the possible factors underlying the retrospective attribution of willpower as an important component in achieving abstinence, the researchers examined non-treatment-related influences and processes that coincided with the alcoholics’ development of abstinence. From these analyses, which were primarily based on self-reports by the study participants and therefore may contain some bias, the researchers identified four factors associated with stable abstinence.

First, approximately two-thirds of the stably abstinent alcoholics developed some form of substitute dependency. These substitute dependencies had many different forms, ranging from overeating, chain smoking, or using tranquilizers to compulsively working, depending excessively on one’s parents, or becoming strongly involved in either AA or a religious group.

Second, abstinence was reinforced by events contingent on alcohol use that systematically altered the consequences of alcohol consumption (i.e., behavior modification) and thus constantly reminded the alcoholic that alcohol was an “enemy.” These behavior modifications took the form of medical consequences (e.g., painful stomach problems after alcohol consumption), legal consequences (e.g., probation requirements), or social supervision and sanctions (e.g., employers who threatened job loss or spouses who threatened divorce in cases of relapse).

Third, enhanced hope, self-esteem, or both assisted the alcoholic in maintaining abstinence. Both evangelical religious involvement and AA participation served as sources of hope and self-esteem. Such intense involvement may have provided group forgiveness and relieved the feelings of shame over past relapses and over the negative impact on others caused by these relapses.

Fourth, abstinence often was associated with the development of new love relationships (e.g., with a new spouse or mentor). In contrast, renewal of existing relationships—for example, through marriage therapy—was less effective (Orford and Edwards 1977), possibly because the emotional wounds inflicted by the alcoholic on his family members and on himself were too deep to be overcome permanently.

Self-help organizations, such as AA, can provide all four of the non-treatment-related factors associated with abstinence. However, by employing these four factors themselves, alcoholics also can achieve abstinence outside of AA. Thus, most of the stably abstinent Core City alcoholics used either AA or at least two of the four factors outside of AA. In general, AA membership was more commonly used as a means of achieving abstinence when the severity of the men’s alcohol-related problems increased.

The four non-treatment-related factors also form the basis of some relapse prevention programs that use cognitive-behavioral techniques (e.g., providing positive feedback for successful abstinence, recalling alcohol-related negative experiences, and avoiding relapse-provoking situations) to sustain abstinence (e.g., Marlatt and Gordon 1985). Components of these programs include changing the alcoholic’s perception of alcohol from positive to negative, developing a plan to stop drinking that enlists the help of others, developing ways of recognizing an imminent relapse and coping with the situation, developing social supports that help reinforce sobriety, and providing substitutes for drinking.

### Stability of Abstinence

An important question in alcoholism treatment is how long abstinence must last before recovery can be considered secure. Many treatment outcome studies follow their subjects for only 6 to 12 months. Longterm followup of both the Core City and College samples demonstrated, however, that relapse rates were still high (41 percent) after 2 years of abstinence but fell dramatically after additional years of abstinence. For example, only 7 percent of the men who had achieved 6 years of abstinence eventually relapsed; the documented abstinence duration of men with at least 6 years of abstinence who never relapsed ranged from 9 to 33 years (with a mean of 20 years). These findings indicate that a 6- to 12-month followup probably is not sufficient to evaluate the efficacy of a specific treatment in inducing long-term recovery.

### Consequences of Abstinence

AA and most other treatment programs generally consider abstinence the only desirable treatment outcome. The effects of abstinence on the alcoholic’s physical and psychological well-being, however, rarely have been examined. A comparison of the progressive alcoholics, stably abstinent alcoholics, and nonalcoholics in the Core City and College samples demonstrated that abstinence does not automatically restore an alcoholic’s physical and psychological health. For example, the short-term death rate among the abstinent alcoholics in the College sample was similar to that among the progressive alcoholics. In addition, compared with nonalcoholics, the stably abstinent Core City men still manifested an increased death rate. Nevertheless, long-term followup of the Core City men demonstrated that physical health steadily improved among the stably abstinent men compared with the chronic alcoholics.

Similarly, abstinence improved the psychological health and quality of life of the securely abstinent Core City alcoholics. For example, the stably abstinent men exhibited significantly lower levels of psychiatric disabilities than the progressive alcoholics and more closely resembled the nonalcoholic men in the sample. Similarly, the stably abstinent men were comparable with the nonalcoholics in their enjoyment of their marriages and family lives and in their occupational success.

Nonetheless, the beneficial effects of abstinence may take several years to develop. For example, Kurtines and colleagues (1978) found that newly abstinent alcoholics were “less normal” on several measures of psychological functioning than alcoholics who had been abstinent for more than 4 years. Similarly, Core City subjects who had been abstinent for less than 3 years had higher levels of psychopathology and more closely resembled active alcoholics than did subjects who had been abstinent for more than 3 years (at an average of 10 years). Divorce and depression are common during the early recovery stages: Newly abstinent alcoholics must readjust to their familial responsibilities and occupational roles. These adjustments can be stressful and time consuming, especially in patients with more severe and prolonged alcoholism. Accordingly, abstinence should not be considered a goal in itself but a means to achieve overall social rehabilitation.

### Return to Asymptomatic Drinking

Several researchers have suggested that successful treatment outcomes for alcoholics include not only abstinence but also a return to asymptomatic drinking (Edwards and Grant 1980; Sobell and Sobell 1978). Among the 111 alcoholic Core City subjects, 42 had returned to asymptomatic drinking (i.e., drinking more than once a month for at least 2 years without experiencing any problems) at age 47. Twenty-two of these men had been asymptomatic for more than 3 years. Compared with the men who remained alcoholic or who became abstinent, the asymptomatic drinkers had experienced significantly fewer alcoholism symptoms, were less likely to have been alcohol dependent, and had fewer alcohol-related job or medical problems. In this sense, they resembled people with high blood pressure or diabetes, who can control their illness by life changes and diet.

Further followup demonstrated, however, that even in these subjects, a return to controlled drinking only rarely was a stable outcome. At age 60, only 6 of the 42 men could still be considered stable asymptomatic drinkers; the others had either relapsed, become stably abstinent, left the study, or had been reclassified as not meeting the criteria for alcohol abuse. In contrast, the long-term outcome was more stable for Core City subjects who at age 47 had been abstinent for more than 3 years. At followup 15 years later, almost all of these men were still abstinent, and only one man had relapsed.

These findings indicate that although a short- to midterm return to controlled drinking is possible for many alcoholics, a long-term return to controlled drinking is a rare and unstable outcome. Similarly, Helzer and colleagues (1985) found that among 1,289 clinically treated alcoholics, only 1 to 2 percent returned to asymptomatic drinking for more than 1 or 2 years. Consequently, abstinence may be a more useful therapeutic focus for the vast majority of alcoholics than an effort to return to asymptomatic drinking. The exception to this generalization is that people in the early stages of alcohol abuse might be offered a monitored trial of controlled drinking (Sanchez-Craig and Lei 1986) in the same way that a trial of weight reduction might be offered to someone with borderline hypertension or diabetes before prescribing medication.

## Summary

The prospective, longitudinal studies of both the Core City and College samples described in this article have helped researchers and clinicians to begin finding answers to the five questions listed earlier. Analyses of the temporal order in which alcoholism and other psychiatric disorders, such as depression and anxiety, developed in the study subjects strongly suggest that these disorders developed as consequences of alcoholism and that alcoholism in most patients was not secondary to other psychiatric disorders. The only exception to this rule appeared to be sociopathy, which constitutes a risk factor for the later development of alcoholism. Other risk factors for alcoholism identified in these studies included cultural factors (i.e., a person’s ethnic background) and genetic factors (i.e., a family history of alcoholism). These characteristics may help distinguish people who eventually become alcoholic from those who do not. Conversely, the childhood environment—beyond the influences of an alcoholic parent—did not appear to contribute to the risk of alcoholism.

The longitudinal evaluation of the study subjects demonstrated that the development and prognosis of alcoholism can vary significantly among individual drinkers. For example, while some people (e.g., sociopaths) developed the symptoms of alcoholism after only several months of heavy drinking, other “late onset” alcoholics drank heavily for many years before becoming alcoholic. Similarly, alcoholism did not always progress inexorably; in some subjects it remained chronic for decades without either progressing or improving. The subjects’ long-term prognoses did not appear to depend on whether they received treatment or what the treatment entailed. Rather, recovery rates of 2 to 3 percent per year were observed in both treated and untreated subjects. AA appeared to be at least as effective as clinic treatment in helping alcoholics to begin stable abstinence; however, it was more important for maintaining stable recovery, partly because, like treatment for other chronic disorders, AA is used daily or weekly for years. Finally, long-term followup of the Core City and College samples demonstrated that (1) for most alcoholics, abstinence was the most effective treatment goal and (2) a return to controlled drinking, although feasible in some alcoholics (especially those at early stages of alcohol abuse), generally resulted in an unstable outcome.

## Footnotes

1 Alcohol-related problems contained in the Problem Drinking Scale include complaints by friends and family members about a person’s drinking behavior, marital problems, multiple job loss, alcohol-related arrests, and visits to either clinics or Alcoholics Anonymous for alcoholism treatment.

2 In children and adolescents, sociopathy is characterized by behaviors such as juvenile delinquency, truancy, and school behavioral problems.
